# Breast cancer screening practices and associated factors among Chinese‐Australian women living in Sydney: A cross‐sectional survey study

**DOI:** 10.1111/nhs.12925

**Published:** 2022-02-28

**Authors:** Lei Wang, Lynette Mackenzie, Zakia Hossain

**Affiliations:** ^1^ School of Rehabilitation Kunming Medical University Kunming China; ^2^ Discipline of Occupational Therapy, School of Health Sciences The University of Sydney Sydney New South Wales Australia; ^3^ School of Health Sciences University of Sydney Sydney New South Wales Australia

**Keywords:** barriers, clinical breast examination, cross‐cultural health, early detection, mammogram, screening

## Abstract

This study aimed to investigate breast cancer screening practices and associated factors among Chinese‐Australian women. A cross‐sectional quantitative survey method including convenience and snowball sampling was used to recruit 115 Chinese‐Australian women living in Sydney, using a self‐administered survey. In all, 69.8% of participants reported recent clinical breast examinations and 73.3% had mammograms. Age, religion, employment status, and length of residence were associated with having a clinical breast examination. Income was related to having a mammogram. Associations between knowledge of breast cancer, cancer‐related beliefs, and screening participation were found. Length of residence in Australia was the strongest predictor of having a clinical breast examination and mammogram. The most common barrier to mammography was if women felt that doctors did not recommend it to them. Chinese‐Australian women need to be educated about awareness of their usual breast health to be aware of any changes, especially if women are not eligible for mammography or have difficulty in accessing health services. Tailored programs, improving screening experiences, and minimizing perceived barriers are needed to promote early detection of breast cancer among Chinese–Australian women.

**Key points**

- Early detection of breast cancer through screening is an important strategy to improve breast cancer outcomes.
- Little is known about the current breast cancer screening practices of Chinese‐Australian women who are at increased risk of breast cancer upon migration to Australia.
- Chinese‐Australian women are under‐represented in breast cancer screening and there are barriers to participation in screening.

## 1 INTRODUCTION

In Australia, early detection plays a leading role in reducing mortality from breast cancer; however, little is known about how Chinese‐Australian women engage in breast cancer screening. Breast cancer is the most common invasive cancer and the second leading cause of death from cancer among women in Australia (Cancer Australia, 2021). Due to the high incidence of breast cancer, early detection through breast cancer screening plays a vital role in early diagnosis and survival of breast cancer (Jin, 2014). When cancers are detected early, they are easier to treat, and the chances of survival are higher.

In Australia, a national breast‐screening program has been established to provide free mammographic screening biannually for asymptomatic women aged 40 and over (Australian Institute of Health and Welfare, 2019a; Breast Cancer Network Australia, n.d.). Cancer Australia (2015) recommends that women who are not attending routine mammograms may benefit from regular clinical breast examination (CBE) and women of all ages are encouraged to be aware of the normal look and feel of their breasts and to report any unusual breast changes to their general practitioner (GP). However, the Australian Institute of Health and Welfare (2019b) reported that women from a culturally or linguistically diverse background aged 50 to 69 and who speak a language other than English at home have a lower participation rate (49%) in the national screening program than other Australian women (55%). There is limited current literature exploring the breast cancer screening practices among women from different cultural and linguistic backgrounds in Australia. This study aims to focus on Chinese‐Australian women and explore their practices in relation to breast cancer screening.

The Chinese‐Australian population is one of the fastest‐growing in Australia and doubled from 2008 to 2018 (Australian Department of Home Affairs, 2020). Migrants born in China are the second largest group of overseas‐born Australians and the largest non‐English‐speaking ethnic group in Australia (Australian Bureau of Statistics, 2019a). Although the incidence of breast cancer among Chinese‐born women is lower than Australian‐born women (Weber et al., 2014), the risk of breast cancer is increased upon migration to Australia and breast cancer remains the primary cause of death among Chinese women following migration to Australia (Levesque et al., 2020). Additionally, Chinese‐Australian women have a higher incidence of breast cancer than Chinese women living in China (Supramaniam et al., 2006). Therefore, investigating the breast cancer screening practices of Chinese‐Australian women who are migrants to Australia is important.

Most of the available data about breast cancer screening practices among Chinese migrant women is based on older overseas studies. These studies found that Chinese women have an increased risk of developing breast cancer following their resettlement in Western countries; however, they were less likely to undertake breast cancer screening (Kwok et al., 2007; Wu et al., 2008). One Canadian study also found that the low uptake of breast cancer screening among Chinese migrant women could be associated with demographic and acculturation factors and Chinese cultural beliefs and attitudes (Tu et al., 2005). In that study, age, employment status, and marital status were associated with participation in mammography among Canadian Chinese women. More generally, another Canadian study found that breast cancer screening barriers like language, acculturation limitations, and knowledge deficit should be addressed with all migrant groups (Vahabi et al., 2015). Some other international studies suggested that fluency in spoken English and length of residence had a positive impact on breast cancer screening (Todd et al., 2011). However, English fluency has not been associated with mammographic screening (Lee‐Lin et al., 2012) and participation in breast cancer screening may be inhibited by Chinese traditional cultural beliefs (Liang et al., 2009). Some international findings may not be applicable due to differences within the Australian health care system, such as efforts in promoting breast cancer screening and the immigration history of Australia for Chinese‐Australian women.

In Australia, there are no national data regarding the participation rate in breast cancer screening among Chinese‐Australian women. Only two quantitative studies have reported participation rates of breast cancer screening among Chinese‐Australian women. In a sample of Chinese‐Australian women living in Sydney, 35.4% of those aged 40 years and over had a CBE annually and 75% of those aged 50–69 years had a mammogram biannually (Kwok et al., 2012a). Furthermore, English proficiency, length of stay, level of education, and employment status were not associated with screening. However, screening was related to positive attitudes toward health checkups and the perception of fewer barriers to screening (Kwok et al., 2012a, 2012b). Given the importance of early detection of breast cancer, there is a need to investigate the current breast cancer screening practices of Chinese‐Australian women and to fully understand the factors associated with uptake of screening as well as any barriers regarding their screening participation.

Therefore, this study aimed to (i) investigate the practices of Chinese‐Australian women living in Sydney in relation to the main breast cancer screening tests (CBE and mammography); and (ii) identify any factors associated with their breast cancer screening behaviors.

## 2 METHOD

This cross‐sectional quantitative study used self‐administered surveys for data collection from July to November 2016. The study was approved by the University of Sydney Human Research Ethics Committee (approval number: 2015/295). There was a potential for the survey questions to be sensitive, so participants were approached in person and the first author also provided an oral explanation in Mandarin (oral Chinese) before participants completed the survey. During the initial contact, potential participants were given a Participant Information Statement (PIS), a consent form, and a copy of the study survey in English or Simplified Chinese (written Chinese). All participants were asked to sign the consent form before completing the survey. Participants were able to choose to return the signed consent form and the study survey immediately or to send them to the chief investigator's office using stamped addressed envelopes.

The target population for the study were Chinese‐Australian women living in Sydney. For this study, the term Chinese‐Australian women refers to any Chinese woman who migrated to Australia from mainland China or Hong Kong. The eligibility criteria included Chinese‐Australian women who were (i) aged 35 years and over, as breast cancer prevalence starts to increase at this age (Australian Institute of Health and Welfare, 2019b); (ii) living in Sydney for more than 1 year following arrival to target more permanent migration; (iii) able to read simplified Chinese; (iv) able to communicate either in Mandarin or English.

Both convenience and snowball sampling were used to recruit eligible participants and 115 Chinese‐Australian women were recruited through churches, Buddhist organizations, social and community centers, seniors' clubs, and personal networks of the authors in Sydney. The first author is fluent in both Mandarin and English and invited Chinese‐Australian women to participate by attending meetings and events. The choice of these organizations allowed participant recruitment to involve Chinese women from diverse backgrounds to increase study heterogeneity (Tu et al., 2005).

The original survey was first developed in English and was used in a previous breast screening study for Southeast Asian migrant women, and was adapted for this study (Robinson et al., 2016). Some items were modified for Chinese migrant women. For instance, “Taoist” and “no religion” were included in the options of the religion question; the options of “Mandarin” and “Cantonese” were added to the question about language spoken at home; the question of “Were you born in Australia?” was changed to the question of “Where were you born?” with “Mainland China,” “Hong Kong,” and “Taiwan” as options. The modified survey was translated into simplified Chinese by a professional translator and back translation into English was conducted to check that the translation was accurate. The final version was proofread and piloted by the first author who is bilingual in Chinese and English to determine if the meanings were accurate.

The survey had 57 questions. Section one covered sociodemographic background (14 questions including age, religion, education, employment, marital status, income, and acculturation factors such as years of residence in Australia, place of birth, self‐rated English proficiency, language spoken at home, and residence in Sydney). Section 2 covered knowledge and beliefs regarding breast cancer (6 questions including symptoms of breast cancer, treatment of breast cancer, detection of breast cancer, and cancer‐related beliefs). Section 3 covered knowledge and practice of mammography and other clinical practices (12 questions). Section 4 covered any history of breast cancer (7 questions). Section 5 covered personal health (10 questions), and Section 6 covered concerns about breast cancer and barriers to screening (8 questions). The Health Belief Model was used to guide the survey design as this model explains health behavior and the underlying processes involved in decision‐making on health issues such as barriers, perceived susceptibility, and perceived effectiveness (Jones et al., 2014). For instance, survey questions about concerns regarding cancer were related to the perceived susceptibility of breast cancer, and the perceived seriousness of breast cancer. The self‐administered survey took approximately 25 minutes to complete. An English copy of the survey is provided as a Supplementary File in the Supporting Information.

Participants' knowledge of breast cancer was assessed on symptoms of breast cancer (8 items), treatment options for breast cancer (5 items), and detection of breast cancer (5 items). Responses to all items were “yes” or “no.” To assess the degree of knowledge regarding breast cancer, knowledge scales were constructed in SPSS 22.0 on the basis of two items: knowledge of breast cancer symptoms and knowledge of breast cancer treatment options. For symptom knowledge, participants were asked to tick symptoms they knew of which consisted of seven possible responses. If the participant ticked an item indicating that they knew it was a symptom of breast cancer it was coded “1,” and not knowing it was coded “0.” The knowledge scale gave the mean for each group with 0 (not knowing any symptoms) being the lowest possible score and 7 (knowing all symptoms) being the highest possible. For treatment options there were five possible answers, therefore 5 was the highest possible score that could be obtained (knowing all treatment options). Each question also included a “not sure” option, which was excluded from the knowledge scale. Reliability statistics were calculated for each scale. The Cronbach's alpha coefficient was 0.78 for the breast cancer symptom scale (Mean = 3.66, SD = 2.47, *n* = 113), 0.78 for the breast cancer treatment scale (Mean = 2.46, SD = 1.65, *n* = 113), and 0.72 for the breast cancer detection scale (Mean = 2.90, SD = 1.63, *n* = 96).

Survey data were coded and entered into the Statistical Package for Social Sciences (SPSS) version 22.0. All data provided by the participants were entered, regardless of any missing items that were not answered. Descriptive statistics were used to summarize the demographic characteristics and breast cancer screening practices of the participants. Normality was assessed by visual inspection of histograms. The income survey items were recoded into two groups (“$20 000 to $50 000” and “>$50 000”). This was based on the known median family income of new migrants ($27 406) and long‐ term migrants ($53 999) (Australian Bureau of Statistics, 2019b). Based on these figures and the frequency distribution of income categories, it was decided to group them into two, $20 000–50 000 as low and middle income and >$50 k as high‐income categories.

Educational level items were recoded to “Secondary school or less” and “Tertiary”; length of stay in Australia was re‐coded to “<10 years,” “10 to 20 years,” and “>20 years.” All significance levels were set at *P* = 0.05. Both parametric and non‐parametric tests were undertaken to explore the factors related to breast cancer screening practices. Bivariate analysis was conducted to examine the relationship between independent variables (including demographic and acculturation variables, breast cancer knowledge, and cancer‐related beliefs) and dependent variables (screening practices such as breast self‐examination, CBE, and mammogram). Associated variables from this analysis were entered into a binary logistic regression to assess the impact of the predictors on the likelihood of participating in breast cancer screening tests.

Chi‐square tests were conducted to examine the relationship between categorical variables (demographic and acculturation variables) and screening practices. Independent sample *t*‐tests also were used to explore the relationship between the knowledge scales of breast cancer and breast cancer screening practices. The Mann–Whitney *U*‐test and independent sample *t*‐tests were used to analyze the association between cancer‐related beliefs and breast screening participation. Multivariate analysis (the binary logistic regression) was undertaken to predict the dependent variables. Lastly, descriptive statistics were used to summarize the barriers of participating in CBE and mammograms.

## 3 RESULTS

A total of 115 eligible Chinese‐Australian women completed and returned a survey. The characteristics of the participants are shown in Table 1. The age of the participants ranged from 35 to 85 years (mean = 56.5 years, 95% CI: 53.24–59.77), with 62.6% of women aged 50 years and over. Most women were married (79.8%) and had children (92%), were born in Mainland China (88.7%) and spoke Mandarin at home (77.2%). In all, 54.8% were not satisfied with their English ability, consisting of 21.2% who could not speak English and 33.6% who had poor self‐rated English ability. Most participants (61.2%) had lived in Australian for more than 10 years, with a mean length of residence of 14 years. Nearly half of participants indicated they had no religion.

**TABLE 1:** Socio‐demographic characteristics of participants (*N* = 115)

Characteristics	*n*	%
Age (years) (Mean: 56.51; 95% CI: 53.24–59.77)		
35–39	25	21.7
40–49	18	15.7
50–59	17	14.8
60–69	23	20.0
70+	32	27.8
Religion (*n* = 112)^a^		
Buddhist	29	25.9
Christian	30	26.8
No religion	53	47.3
Educational level		
Secondary school or less	38	33.0
Tertiary	77	67.0
Employment status (*n* = 114)^a^		
Unemployed	15	13.2
Working full‐time	45	39.5
Working part‐time	16	14.0
Retired	38	33.3
Number of years employed (Mean: 24.01, 95% CI: 21.69–26.33)		
Marital status (*n* = 114)	#	#
Single	9	7.9
Married	91	79.8
Divorced/ Widowed	14	12.3
Number of children (*n* = 88)^a^		
None	7	8
1	35	39.8
2	33	37.5
3 +	13	14.7
Annual income (*n* = 89)^a^		
$20 000 ‐ $50 000	37	41.6
>$50 000	52	58.4
Years of residence in Australia (*n* = 111)^a^ (Mean:13.95; 95% CI: 12.31–15.60)		
<10 years	43	38.7
10 to 20 years	43	38.7
>20 years	25	22.6
Place of birth		
Mainland China	102	88.7
Hong Kong	13	11.3
Self‐rated English proficiency (*n* = 113)^a^		
Cannot speak English	24	21.2
Poor	38	33.6
Satisfactory	32	28.3
Fluent	19	16.9
Language spoken at home (*n* = 114)^a^		
Mandarin	88	77.2
Cantonese	22	19.3
English	4	3.5
Residential suburb (*n* = 111)^a^		
North Sydney	25	22.5
South Sydney	33	29.7
West Sydney	13	11.7
Inner Sydney	40	36.0

Abbreviation: CI, confidence interval.

^a^ 0 cells (0.0%) have expected count less than 5.

^#^= missing data.

Overall, few participants had experience of breast cancer. No participants reported having had breast cancer themselves and eight (7.4%) reported having had a family member with breast cancer (mother, *n* = 3 a; sister, *n* = 2; and aunt, *n* = 1). One participant reported a death in the family due to breast cancer. In terms of self‐rated health, 17.8% rated their health as excellent, 29.9% as good, 46.7% as satisfactory, 4.6% as poor, and 1% as very poor, which is lower than the Australian mean of 56% for excellent and good ratings combined (Australian Institute of Health and Welfare, 2018). Participants also reported chronic health problems such as high blood pressure (26%), diabetes (10%), heart disease (6%), female reproductive issues (6%), asthma (2%), and other cancer (2%). Over 90% reported having heard of mammograms but only 64.2% indicated they had one in the last two years. In all, 58.3% of participants had completed a CBE within the last year, leaving a proportion of women not adhering to recommendations such as a yearly CBE or biannual mammograms.

Table 2 examines the relationship between categorical variables and breast cancer screening practices. The number of children, language spoken at home, suburb lived in, and place of birth were not significantly related to having a CBE or mammogram. Neither educational level nor level of annual income were significantly associated with having a CBE. However, women were less likely to have a CBE when they were 70 years or older, if they were Buddhist, or were retired. Age group, religion, educational level, and employment status were not significantly related to receiving a mammogram. However, there was a significant association (*P* < 0.05) between the level of annual income and having a mammogram, suggesting that women with a lower income level were more likely to participate in mammographic screening. Neither English proficiency nor length of residence was significantly related to having a mammogram, although a significant association (*P* < 0.05) was found between length of residence and having a CBE.

**TABLE 2:** Association between sociodemographic factors and clinical breast examination (CBE) and mammogram

Factors	Clinical breast examination (CBE) (*n* = 106)				
	No *n* (%)	Yes *n* (%)	*P*	Pearson’s χ^2^	df
**Demographic factors**					
Age					
35–39	5 (22.7)	17(77.3)	<0.001	22.995^a^	4
40–49	5 (29.4)	12(70.6)			
50–59	0 (0.0)	16(100.0)			
60–69	4 (18.2)	18(81.8)			
70+	18 (62.1)	11(37.9)			
Religion (*n* = 103)^#^					
Buddhist	14(53.8)	12(46.2)	0.015	8.439^a^	2
Christian	7(24.1)	22(75.9)			
No religion	11(22.9)	37(77.1)			
Employment status (*n* = 101)^#^					
Unemployed	3(23.1)	10(76.9)	0.016	8.328^a^	2
Working	11(20.0)	44(80.0)			
Retired	16(48.5)	17(51.5)			
**Acculturation factors**					
English proficiency (*n* = 105)^#^					
Cannot speak English	10(43.5)	13(56.5)	0.343	3.334^a^	3
Poor	10(29.4)	24(70.6)			
Satisfactory	6(20.7)	23(79.3)			
Fluent	5(26.3)	14(73.7)			
Years of residence in Australia (n = 102)^#^					
<10 years	18(45.0)	22(55.0)	0.030	6.980^a^	2
10 to 20 years	9(23.7)	29(76.3)			
>20 years	4(16.7)	20(83.3)			
	**Mammogram (n = 90)**				
**Demographic factors**					
Age (*n* = 82)^#^					
35–49	12(41.4)	17(58.6)	0.064	5.487^a^	2
50–69	5(15.2)	28(84.8)			
70+	7(25.0)	13(75.0)			
Annual income (*n* = 66)^#^					
$20 000–50 000	5(17.2)	24(82.8)	0.041	4.179^a^	1
>$50 000	15 (40.5)	22(59.5)			
**Acculturation factors**					
English proficiency (*n* = 89)^#^					
Cannot speak English	5(26.3)	14(73.7)	0.542	2.150^a^	3
Poor	6(20.0)	24(80.0)	#	#	#
Satisfactory	6(24.0)	19(76.0)	#	#	#
Fluent	6(40.0)	9(60.0)	#	#	#
Years of residence in Australia (*n* = 77)^#^					
<10 years	10(32.3)	21(67.7)	0.437	1.657^a^	2
10 to 20 years	10(30.3)	23(69.7)	#	#	#
>20 years	4(17.4)	19(82.6)	#	#	#

Abbreviation: df = degrees of freedom.

# = missing data.

a 0 cells (0.0%) have expected count less than 5.

Knowledge of breast cancer (symptoms, treatment options, and detection of breast cancer) were examined by descriptive statistics. The most common symptom of breast cancer known by participants was a lump (77.0%), followed by nipple discharge (55.8%), swollen underarms (54.0%), changed size or shape of breasts (52.2%), and redness or dimpling of breast (46.0%). For the treatment options of breast cancer, 77.9% of participants identified surgery, and more than half of them identified chemotherapy (53.1%) and radiation therapy (52.2%). Mammography (66.7%) was the first option identified for detecting breast cancer, then CBE and needle biopsy.

Independent sample *t*‐tests were used to assess the relationship between breast cancer knowledge and breast cancer screening practices (CBE and mammogram; Table 3). There were no significant associations between breast cancer knowledge and having a CBE, or knowledge of treatment and knowledge of detection and having a mammogram. Participants who had more knowledge about symptoms of breast cancer were less likely to have a mammogram.

**TABLE 3:** Association between breast cancer knowledge and breast cancer screening practices among participants who had ever performed CBE or mammogram

Breast cancer screening practices	Breast cancer knowledge		
	Symptoms of breast cancer	Treatment of breast cancer	Detection of breast cancer
	*n* mean (SD)	*n* mean (SD)	*n* mean (SD)
**CBE (*n* = 106)**			
Yes	73 4.00 (2.36)	73 2.62 (1.64)	63 3.14 (1.69)
No	31 3.55 (2.47)	31 2.39 (1.59)	25 2.56 (1.45)
Significance	*p* = 0.381 (*t* = 0.88, df = 102)	*p* = 0.511 (*t* = 0.66, df = 102)	*p* = 0.133(*t* = 1.52, df = 86)
**Mammogram (*n* = 90)**			
Yes	65 3.54 (2.39)	65 2.20 (1.69)	55 2.67 (1.72)
No	23 4.91 (2.23)	23 3.00 (1.60)	20 3.40 (1.60)
Significance	*p* = 0.018 (t = −2.41, df = 86)	*p* = 0.051 (t = −1.98, df = 86)	*p* = 0.104 (t = −1.65, df = 73)

Abbreviations: df, degrees of freedom; *t*, Student's *t* statistic.

Most women (94.5%) believed that “cancers are caused by an unhealthy lifestyle.” This belief was not significantly associated with a CBE or mammogram, but the population who believed this were more likely to participate in CBE and mammograms (Table 4).

**TABLE 4:** Association between cancer‐related beliefs and the breast cancer screening practices among participants who had ever performed breast screening

Cancer‐related beliefs	Clinical breast examination (CBE) (*n* = 106)		Mammogram (*n* = 90)	
	No	Yes	No	Yes
	*n* (MR)	*n* (MR)	*n* (MR)	*n* (MR)
Cancers are caused by an unhealthy lifestyle.	6 (40.75)	95 (51.65)	5 (36.80)	81 (43.91)
	*P* = 0.264, U = 223, W = 244		*P* = 0.406, U = 169, W = 184	

	No	Yes	No	Yes
	*n* (%)	*n* (%)	*n* (%)	*n* (%)
Cancer is hereditary.				
No	16 (47.1)	18 (52.9)	7 (22.6)	24 (77.4)
Yes	15 (25.0)	45 (75.0)	14 (29.2)	34 (70.8)
	*P* = 0.029, χ^2^ = 4.778^a^, df = 1		*P* = 0.518, χ^2^ = 0.419^a^, df = 1	
People get breast cancer as they did not breast feed their babies.				
No	14 (25.9)	40 (74.1)	9 (20.5)	35 (79.5)
Yes	15 (45.5)	18 (54.5)	13 (44.8)	16 (55.2)
	*P* = 0.061,χ^2^ = 3.515^a^,df = 1		*P* = 0.026, χ^2^ = 4.932^a^, df = 1	

Abbreviations: df, degrees of freedom; MR, Mean Rank; U, Mann–Whitney *U*; W, Wilcoxon W. Chi squared analysis.

^a^0 cells (0.0%) have expected count less than 5.

More than half of participants (61.8%) indicated that “cancer is hereditary” and 36.2% of participants indicated that “people got breast cancer as they did not breastfeed their babies.” Women who perceived “cancer is hereditary” were more likely to have a CBE (*P* = 0.029, χ^2^ = 4.778, df = 1), and the women who perceived “people got breast cancer as they did not breastfeed their babies” were less likely to have a mammogram (*P* = 0.026, χ^2^ = 4.932, df = 1).

Binary logistic regression was used to assess the impact of the predictors on the likelihood of participating in breast cancer screening tests. Associated factors (age, years of residence in Australia, religion, employment status, educational level, annual income, symptoms of breast cancer, “Cancer is hereditary” belief, and “People got breast cancer as they didn't breast feed their babies” belief were entered into a binary logistic regression as independent variables. The results are presented in Table 5. The length of residence was the strongest predictor for having a CBE (Wald = 6.14, *P* = 0.013, OR = 1.47), followed by Christian religion (Wald = 4.65, *P* = 0.031, OR = 16.4). This suggested that participants, who stayed in Australia longer and were Christians, were more likely to have a CBE. For having a mammogram, the length of residence also was the strongest predictor (Wald = 5.77, *P* = 0.016, OR = 1.29), followed by age (Wald = 4.28, *P* = 0.039, OR = 0.84) and annual income (Wald = 4.24, *P* = 0.039, OR = 0.03). This indicated that participants, who stayed in Australia longer, were younger, and had lower annual income, were more likely to have a mammogram.

**TABLE 5:** Factors associated with breast cancer screening practices among participants who had ever performed breast screening

	Clinical breast examination (CBE) (*n* = 65)				Mammogram (*n* = 51)			
	W(df)	P	OR	95% CI	W(df)	P	OR	95% CI
Age (years)	0.02(1)	0.88	0.99	(0.86,1.14)	4.28(1)	0.039	0.84	(0.72,0.99)
Years of residence in Australia	6.14(1)	0.013	1.47	(1.08,2.00)	5.77(1)	0.016	1.29	(1.05,1.59)
Religion								
Buddhist (reference)								
Christian	4.65(1)	0.031	16.41	(1.29208.65)	0.58(1)	0.446	0.39	(0.03,4.48)
No religion	3.73(1)	0.053	11.78	(0.96143.9)	0.02(1)	0.904	0.86	(0.08,9.44)
Employment status								
Unemployed (reference)								
Working	0.70(1)	0.403	0.22	(0.01,7.86)	3.30(1)	0.069	0.05	(0.00,1.28)
Retired	2.22(1)	0.136	0.04	(0.00,2.73)	0.02(1)	0.893	0.76	(0.02,38.95)
Educational level								
≤Secondary school (reference)								
Tertiary	0.10(1)	0.757	0.59	(0.02,17.17)	0.44(1)	0.508	2.18	(0.22,21.93)
Annual income								
$20 000–50 000(reference)								
>$50 000	0.07(1)	0.792	0.76	(0.10,5.72)	4.24(1)	0.039	0.03	(0.00,0.84)
Symptoms of breast cancer	1.15(1)	0.283	1.31	(0.80,2.14)	1.89(1)	0.169	0.65	(0.35,1.20)
Cancer is hereditary	0.39(1)	0.531	1.93	(0.25,15.07)	0.00(1)	0.967	0.96	(0.12,7.69)
People got breast cancer as they did not breast feed their babies.	1.28(1)	0.258	0.25	(0.02,2.78)	0.00(1)	0.974	1.04	(0.11,10.12)
Model summary	−2 Log likelihood	Cox & Snell R Square		Nagelkerke R Square	−2 Log likelihood	Cox & Snell R Square		Nagelkerke R Square
	36.387^a^	0.444		0.640	36.749^a^	0.410		0.561

Abbreviations: CI, confidence interval; OR, odds ratio; W, Wald.

Participants indicated there were no problems in having a CBE (60%) or mammogram (69%). However, participants reported higher levels of discomfort (56%), irritation (32%), pain (59%), and embarrassment (29%) with mammograms.

## 4 DISCUSSION

This study investigated the breast cancer screening practices of Chinese‐Australian women in Sydney. The participant group members were fairly typical with a low rate of breast cancer reported, as well as the expected levels of chronic disease and personal health reported. Findings provided an insight into practices and behaviors by examining the associated factors and barriers regarding having a CBE and mammogram. Results indicated that most Chinese‐Australian women had engaged in CBE and mammograms at some stage, but the participation rate in the recommended frequency of breast screening was not high (58.3% for a CBE in the last year and 64.2% for a mammogram in the last 2 years). The participation rate of having a CBE highlighted that most participants had a CBE over a year ago (Kwok et al., 2012b). Women who are not attending routine mammograms may gain benefits from a regular CBE (Cancer Australia, 2015). Therefore, more efforts need to be made to screen women who are not actively targeted by the national mammographic screening program, and GPs are important in promoting CBE practice for women aged under 50 who do not receive an invitation for a biannual mammographic screening.

For women aged between 50 and 69 years, the mammogram screening rate of participants in our study was higher than the national rate for Australian women and women from a culturally or linguistically diverse background (Australian Institute of Health and Welfare, 2019a). Similar findings have been reported elsewhere (Kwok et al., 2012a, 2012b). This indicates that Chinese‐Australian women in this study made good use of mammographic screening and may respond positively to information in the media about the need for and availability of mammograms at this age. It is possible that these relatively high rates of mammography are related to recruitment through Chinese organizations, where women could be exposed to more resources and information. Attending community meetings could also be a significant facilitator to having a mammogram, as group mammography with an interpreter and transportation can help target participants (Kwok et al., 2005).

In our study, Christian women were more likely to have a CBE compared to Buddhist women. This is consistent with a previous study on Chinese‐American women (Tu et al., 2003), and may be explained by the Buddhist concept of karma that encourages people to do morally good things to receive positive consequences and talking about disease or death is discouraged (Licqurish et al., 2017). Therefore, women from the Buddhist religion may not prioritize having a CBE. Our study also found that older women and retired women did not have CBEs perhaps because they may believe that their risks of being diagnosed with breast cancer are low, or that they disliked the more personal experience of a CBE with their doctor compared to a mammogram. Education level and employment status were not associated with participating in mammography screening (Kwok et al., 2012a), although annual income was negatively related to having a mammogram. This may be related to the national mammographic screening program that provides a free biannual mammogram.

Length of residence was the most important predictor for both having a CBE and a mammogram. This is inconsistent with a previous study (Kwok et al., 2012b). Our study suggested that Chinese migrant women who lived in Australia longer were more likely to access a CBE and mammogram. Women who have been in Australia longer may be more familiar with the Australian health service and have more knowledge about breast cancer screening. However, a relationship between English proficiency and practicing screening tests (CBE and mammogram) was not found, which is consistent with two previous studies (Kwok et al., 2012a, 2012b).

Although previous studies indicated that knowledge of breast cancer was not associated with the breast cancer screening behaviors of Chinese migrant women in Australia (Kwok et al., 2012a, 2012b) and the United States (Lee‐Lin et al., 2012), our study found that the scores for knowledge of symptoms of breast cancer were significantly lower for those who had a mammogram. This was an unexpected finding and aligns with the findings of a Spanish study (Pérez‐Lacasta et al., 2019) that suggests when women have more knowledge about the benefits and harms of mammography such as overdiagnosis and false positives they are less likely to participate in breast screening, whereas women without this knowledge were more likely to participate. There may be other motivators for women having a mammogram other than knowing about breast cancer risks. For Chinese women in Malaysia the main motivators to participate in mammography were recommendations from doctors, family and friends (Hassan et al., 2015). This was also found in a population of Singaporean women who indicated that their motivators for mammography were to have control over their health, because of family and friends' encouragement and due to a physician's advice (Teo et al., 2013). This suggests personal recommendations rather than knowledge may be more important.

Most Chinese migrant women reported that there were no problems with undertaking breast cancer screening tests. However, negative experiences involving pain, discomfort, irritation, and embarrassment were reported, particularly for mammograms. These negative experiences may prevent Chinese‐Australian women from having screening tests. Previous studies stated that barriers were significantly related to having a mammogram (Kwok et al., 2012a, 2012b). Despite completion of tertiary education, participants disliked being exposed to unnecessary radiation from mammography. This may be explained by the belief that breast cancer is inevitable (Kwok & Sullivan, 2006) and Chinese cultural beliefs that breast cancer screening is not needed when they are feeling well (Tang et al., 2000) or that breast cancer is perceived as a “white women's disease” (Kwok & White, 2011, p. 89).

Participants in our study may rely on the free mammographic screening rather than being aware of the normal look and feel of their breasts and reporting any unusual breast changes. It is important to provide more information and resources that help Chinese migrant women overcome their barriers to mammograms. Previous studies identified other barriers of having a mammogram in Chinese migrant women, such as language barriers (Kwok & White, 2011; Levesque et al., 2020; Todd et al., 2011), modesty issues, transportation, and embarrassment (Kwok et al., 2012a).

Our findings have shown that breast cancer screening practices need to be continually promoted to Chinese‐Australian women by health professionals, especially for newly arrived migrants. Chinese migrant women who are not connected to community Chinese organizations may also need to be targeted more actively such as providing culturally appropriate information (Kwok & White, 2011), and recognizing that migrants may be vulnerable to depression, anxiety, and stress (Lee et al., 2013). Improving screening experiences and minimizing the perceived barriers can assist to promote the early detection of breast cancer among Chinese‐Australian women. The identified factors associated with breast screening practices in this study are important to developing tailored and culturally sensitive breast screening education programs to meet the unique needs of this population.

### 4.1 Study limitations

This study used a convenience sample and not a representative population sample, and therefore the findings cannot be easily generalized. The use of self‐administered surveys may also lead to some study data (e.g. the screening participation rate, annual income) being overestimated or underestimated by participants, and social desirability bias may be present (van de Mortel, 2008). Survey fatigue may have been a factor due to the length of the survey. Despite the limitations, the study investigated a comprehensive range of factors and recruited key informants to explore Chinese‐Australian women's breast cancer screening practices.

## 5 CONCLUSION

Whilst most Chinese‐Australian women had engaged in CBE and mammograms, they were having mammograms as recommended rather than having a CBE regularly. GPs are essential for encouraging women to have a CBE, especially those who are not yet invited to mammography screening. Some participants describe negative experiences of mammograms such as pain, discomfort, irritation, and embarrassment, which may discourage participation in screening tests. Prior to undertaking CBE or mammography, Chinese‐Australian women need to be educated about awareness of their usual breast health to be aware of any changes, especially if women are not eligible for mammography or have difficulty in accessing health services.

## 6 RELEVANCE FOR CLINICAL PRACTICE

Findings have shown that breast cancer screening practices need to be continually promoted to Chinese‐Australian women by health professionals, especially for those under the age of the National free mammography program, and newly arrived migrants. These findings also demonstrate that the situation has continued over time. Chinese migrant women who are not connected to community Chinese organizations may also need to be targeted more actively such as by providing culturally appropriate information (Kwok & White, 2011), and recognizing that migrants may be vulnerable to depression, anxiety, and stress (Lee et al., 2013). Improving screening experiences and minimizing the perceived barriers can assist in promoting the early detection of breast cancer among Chinese‐Australian women. The identified factors associated with breast screening practices in this study are important to develop tailored and culturally sensitive breast screening education programs to meet the unique needs of this population.

## AUTHOR CONTRIBUTIONS

Study design: Lei Wang, Lynette Mackenzie, Zakia Hossain.

Data collection: Lei Wang, Lynette Mackenzie, Zakia Hossain .

Data analysis: Lei Wang, Lynette Mackenzie, Zakia Hossain .

Manuscript writing: Lei Wang, Lynette Mackenzie, Zakia Hossain .

## Supporting information


**Appendix S1**. Supporting Information.

## Data Availability

The data that support the findings of this study are available on request from the corresponding author. The data are not publicly available due to privacy or ethical restrictions.
