# Supplementary material for: Breast cancer screening practices and associated factors among Chinese‐Australian women living in Sydney: A cross‐sectional survey study
Source: Nurs Health Sci. 2022 Feb 28;24(1):293–303. doi: 10.1111/nhs.12925 (PMC9305288; doi:10.1111/nhs.12925)
Supplement: Supplementary file 1 — Appendix S1. Supporting Information. [file NHS-24-293-s001.docx]

Study Questionnaire in English

ID No.

**Confidential**

**South East Asian (Chinese) Migrant Women’s Breast Cancer Project**

| Breast cancer is the most commonly diagnosed malignant tumour among women in Australia. Studies have shown that early detection of the disease strongly contributes to increased chances of survival, as well as the contribution of ethnic differences in the survival rate of breast cancer. However, there is limited evidence on ethnic differences in the survival rate of breast cancer in Australia, especially in Sydney. Therefore, the purpose of this study is to record the breast cancer screening practices, including breast self examination (BSE), clinical breast examination (CBE) and mammography, of a sample of South East Asian migrant women, in particular Chinese migrant women residing in the Sydney Metropolitan area. This will be achieved by conducting a questionnaire on a sample size of approximately 200 participants.  This questionnaire is divided into six broad sections:   1. Socio-demographic background 2. Knowledge regarding breast cancer 3. Knowledge on and practices of breast self examination, mammography and other clinical practices 4. History of Breast Cancer 5. Personal health 6. Health Belief model for Breast Cancer   This survey plays a crucial role in enabling us to find out the levels of awareness and knowledge of breast cancer and its screening practices among migrant women, in particular Chinese Migrant women in Sydney metropolitan area (SMA).  Your participation in this survey is completely voluntary and you can withdraw from the survey at any time without any penalty. If you decide to participate in this survey please sign the Consent form and complete this questionnaire. Your identity will remain confidential throughout the study.  **We appreciate it if you would answer every question in this questionnaire**. |
| --- |

*Where required, please answer the question by placing a cross (X) the most applicable box (*☐*)*

**Section 1: Socio-demographic Background**

1. Age: ________________________
2. Country of birth ________________________
3. Religion:

- Buddhist ☐
- Christian ☐
- Muslim ☐
- Taoist ☐
- No religion ☐
- Other *(please specify)*  ____________________

1. Highest level of education. ____________ (years)
2. Highest attained level of education:

- No formal education ☐
- Primary school ☐
- Secondary ☐
- Tertiary
- Diploma ☐
- Undergraduate degree ☐
- Postgraduate degree ☐
- Other *(please specify)* ______________________

1. Employment status (main employment):

- Unemployed ☐ *[ go to Question 8]*
- Full time ☐
- Part time ☐
- Casual/Contract ☐
- Other *(please specify)* ______________________

1. Number of years employed ______________ (years)
2. Marital status:

- Married ☐
- Widowed ☐
- De facto ☐
- Divorced/Separated ☐
- Single ☐ *(Go to question 10)*

1. If you have children, how many? ________________________
2. What is your (if you are single) / your family’s (combined you, your partner and/or children’s income) **annual income**?
   - $20,000 - $50,000 ☐
   - $50,000 - $100,000 ☐
   - $100,000 - $150,000 ☐
   - $150,000 -$200,000 ☐
   - $200,000 + ☐
3. In what year did you migrate to Australia?

- 1. _________________(year)
  2. Where were you born?
- Mainland China ☐
- Hong Kong ☐
- Tai Wan ☐
- Other *(please specify)* ______________________

1. How do you rate your English-speaking ability?

- Cannot speak English ☐
- Poor ☐
- Satisfactory ☐
- Fluent ☐
- Very fluent ☐

1. What language do you speak at home?

- Mandarin ☐
- Cantonese ☐
- English ☐
- Others *(please specify)* __________________

1. What is the area of the suburb you live currently?

Area ___________________________（for example: Ashfield）

**Section 2: Knowledge and beliefs regarding Breast Cancer**

1. Have you heard of breast cancer?

- Yes ☐
- No ☐

1. What are the symptoms of Breast Cancer? (You can choose more than one from the following.)

- Lump ☐
- Changed size or shape of breasts ☐
- Nipple discharge ☐
- Crusting, ulcer or redness of nipples ☐
- Redness or dimpling of breast ☐
- Swollen underarms ☐
- Breast swelling ☐
- Not sure ☐

1. If you discover any of the above, how soon should you seek help?

- Immediately ☐
- Within 1-3 months ☐
- After 3 months ☐
- Not at all ☐
- Not sure ☐

1. What are the treatment options available for breast cancer? (You can choose more than one from the following.)

- Prescription drugs ☐
- Chemotherapy ☐
- Surgery ☐
- Radiation therapy ☐
- Hormone therapy ☐
- Don’t know ☐

1. Do you believe there is anything you can do to prevent getting cancer?

- No ☐
- Yes ☐
- Not sure ☐

1. Why do you think people get **cancer**? (Please answer yes or no for each)

- It is a God’s punishment.  No☐ Yes ☐
- It is fate. No☐ Yes ☐
- It is bad luck. No☐ Yes ☐
- Cancer is a contagious disease. No☐ Yes ☐
- Cancer is hereditary.   No☐ Yes ☐
- They have an unhealthy lifestyle. No☐ Yes ☐

(Such as no exercise, smoking or eating unhealthy food)

- They do not breast feed their babies. No☐ Yes ☐

**Section 3: Knowledge and practice of mammography and other clinical practices**

1. Have you ever had a breast examination by a medical professional?

- Yes ☐
- No ☐ *(go to question 25)*

1. Which health professional performed the clinical breast examination in the last 2 years?

- GP ☐
- Nurse ☐
- Both ☐
- Other (Please specify) ____________________________

1. When was your last clinical breast examination performed?

- Within the last week ☐
- Within the last month ☐
- Within the last 3 months ☐
- Within the last 6 months ☐
- Within the last year ☐
- Over a year ago ☐

1. How did you find the experience of having a clinical breast examination?
   *Place a cross (X) in the most applicable box (*☐)

*SA=Strongly Agree A=Agree N= Neutral D=Disagree SD= Strongly Disagree*

- Painful

SA ☐ A ☐ N ☐ D ☐ SD ☐

- No problem

SA ☐ A ☐ N ☐ D ☐ SD ☐

- Uncomfortable

SA ☐ A ☐ N ☐ D ☐ SD ☐

- Irritating

SA ☐ A ☐ N ☐ D ☐ SD ☐

- Embarrassing

SA ☐ A ☐ N ☐ D ☐ SD ☐

1. Have you heard about mammogram?

- Yes ☐
- No ☐ *(go to question 33)*

1. Where did you find the information about mammograms? (You can choose more than one from the following.)

- GP ☐
- Breast cancer NSW invitation ☐
- Media (TV/radio/Newspapers/magazines) ☐
- Internet ☐
- Community health centres ☐
- Relative/friends ☐
- Others (Please Specify) _________________________

1. How often should a mammogram be performed?

- Every 2 years ☐
- Every 5 years ☐
- Every 10 years ☐
- Not sure ☐

1. Have you ever had a mammogram?

- Yes ☐
- No ☐ *(Go to question* ***31****)*

1. When was the last time you had a mammogram?

- Within the last 2 years ☐
- Within the last 5 years ☐
- Over 5 years ago ☐

1. How did you find the experience of having a mammogram?
   *Place a cross (X) in the most applicable box (*☐)

*SA=Strongly Agree A=Agree N= Neutral D=Disagree SD= Strongly Disagree*

- Painful

SA ☐ A ☐ N ☐ D ☐ SD ☐

- No problem

SA ☐ A ☐ N ☐ D ☐ SD ☐

- Uncomfortable

SA ☐ A ☐ N ☐ D ☐ SD ☐

- Irritating

SA ☐ A ☐ N ☐ D ☐ SD ☐

- Embarrassing

SA ☐ A ☐ N ☐ D ☐ SD ☐

1. What is/are your reason(s) for not having a mammogram? (You can choose more than one from the following.)

- Unaware of this service ☐
- No knowledge about mammogram? ☐
- Too expensive ☐
- Too far away (distance) ☐
- Religious reason ☐
- Other *(please specify) _____________________________________*

1. How can you detect if you have breast cancer? (You can choose more than one from the following.)

- Breast Self Examination (BSE) ☐
- Clinical breast examination ☐
- Mammography ☐
- Ultrasound ☐
- Magnetic Resonance Imagine (MRI) ☐
- Needle biopsy ☐

**Section 4: History of Breast Cancer**

1. Have you ever experienced breast cancer?

- Yes ☐
- No ☐ *(go to question* ***37****)*

1. What was/were the symptoms you have experienced (You can choose more than one from the following.)

- Lumps ☐
- Swelling ☐
- Redness/Inflammation ☐
- Itchiness/Rashes ☐
- Pain during breast feeding ☐
- Nipple discharge ☐

1. Which of the following treatment services did you seek? (You can choose more than one answer from the following.)

- General Practitioner ☐
- Paramedic ☐
- Pharmacy ☐
- Hospital ☐
- Naturopath ☐
- Homeopath ☐
- Oncologist ☐
- Radiologist ☐
- Other *(please specify)* ________________________

1. If you did not seek any treatment, why not?

_______________________________________________________________

_____________________________________________________________

1. Has anyone in your family ever had breast cancer?

- Yes ☐
- No ☐ *(Go to question* ***40****)*

1. If yes, please tick who was in the family affected? (multiple answers)

- Mother ☐
- Sister ☐
- Grandmother ☐
- Aunty ☐
- Others *(Please specify)*__________________________

1. Has there even been a death in the family due to breast cancer?

- Yes ☐
- No ☐

**Section 5: Personal Health**

1. How would you describe your health?

- Excellent ☐
- Good ☐
- Satisfactory ☐
- Poor ☐
- Very poor ☐

1. Have you experienced any of the following in the past 12 months?

- Asthma Yes ☐ No ☐
- Diabetes Yes ☐ No ☐
- Cancer Yes ☐ No ☐
- Heart disease Yes ☐ No ☐
- High blood pressure Yes ☐ No ☐
- Female reproductive problems *(please specify) ________________________________________________________________________*

1. Which of the following health facilities have you used in the past 12 months?

- General Practitioner Yes ☐ No ☐
- Paramedic Yes ☐ No ☐
- Pharmacy Yes ☐ No ☐
- Local hospital Yes ☐ No ☐
- Naturopath Yes ☐ No ☐
- Physiotherapy Yes ☐ No ☐
- Occupational therapy Yes ☐ No ☐
- Radiologist Yes ☐ No ☐
- Chinese medicine Yes ☐ No ☐

1. From the above, which service did you use most?

__________________________________________________

1. The service you used most, how frequent were your visits?

- Once a month ☐
- Quarterly (every 3 months) ☐
- Every 6 months ☐
- Annually ☐
- More than a year ☐

1. If you haven’t used any of the above, what was the reason?

- Unaware of this service ☐
- GP has not recommended ☐
- Unavailable ☐
- Too expensive ☐
- Too far away ☐
- Objection from husband ☐
- No female practitioner available ☐
- No doctor speaks Chinese ☐
- No need ☐
- Other *(please specify)*  ________________________

1. Which of the following apply to you?

- I maintain a healthy well-balanced diet Yes ☐
- I frequently take action to improve my health Yes ☐
- I regularly go for check-ups as required Yes ☐
- I exercise regularly Yes ☐

1. How important are the following factors for you when you consider for a new health practitioner?
   *Place a cross (X) in the most applicable box (*☐)

*SA=Strongly Agree A=Agree N= Neutral D=Disagree SD= Strongly Disagree*

- Language

SA ☐ A ☐ N ☐ D ☐ SD ☐

- Ethnicity

SA ☐ A ☐ N ☐ D ☐ SD ☐

- Gender – female

SA ☐ A ☐ N ☐ D ☐ SD ☐

- Cost

SA ☐ A ☐ N ☐ D ☐ SD ☐

- Convenience (location)

SA ☐ A ☐ N ☐ D ☐ SD ☐

- Religion

SA ☐ A ☐ N ☐ D ☐ SD ☐

- Quality of care

SA ☐ A ☐ N ☐ D ☐ SD ☐

1. Which of the following would you prefer to have a female practitioner:

*HP = High preference SP= Some preference N= Neutral LP= less preference*

*NP = No preference*

- Dental exam:

HP ☐ SP ☐ N ☐ LP ☐ NP ☐

- Physical exam:

HP ☐ SP ☐ N ☐ LP ☐ NP ☐

- Breast exam:

HP ☐ SP ☐ N ☐ LP ☐ NP ☐

- Gynecological exam: e.g. pap smear

HP ☐ SP ☐ N ☐ LP ☐ NP ☐

1. Are there any medical/health services that you would refuse to get from a male practitioner? Please specify ______________________________

**Section 6: Concerns about breast cancer**

(Based on barriers, perceived susceptibility, and perceived effectiveness)

1. When I think about breast cancer, I feel:

- Scared ☐
- Anxious ☐
- Nervous ☐
- Nauseous ☐
- Fine ☐
- Nothing ☐

1. I feel breast cancer will threaten my (multiple answers) :

- Marriage (or significant relationship) ☐
- Career ☐
- Relationship with loved ones ☐
- Financial security ☐

1. My chances of getting breast cancer in the future are:

- Very likely ☐
- Likely ☐
- Probable ☐
- Unlikely ☐
- Very unlikely ☐

1. How likely is it that I am at risk for developing breast cancer due to my:

*Cross (X) the most applicable box*

VL=Very Likely L= Likely M=Maybe U=Unlikely HU=Highly Unlikely

- Age:

VL ☐ L ☐ M ☐ U ☐ HU ☐

- Health:

VL ☐ L ☐ M ☐ U ☐ HU ☐

- Family History:

VL ☐ L ☐ M ☐ U ☐ HU ☐

1. How likely is it that mammogram can:

*Cross (X) the most applicable box*

VL=Very Likely L= Likely M=Maybe U=Unlikely HU=Highly Unlikely

- Be very beneficial for me:

VL ☐ L ☐ M ☐ U ☐ HU ☐

- Help me detect a lump in my breast:

VL ☐ L ☐ M ☐ U ☐ HU ☐

- Help me detect breast cancer before it is too late

VL ☐ L ☐ M ☐ U ☐ HU ☐

- Make me worry less about getting breast cancer:

VL ☐ L ☐ M ☐ U ☐ HU ☐

- Will decrease my chances of dying from breast cancer

VL ☐ L ☐ M ☐ U ☐ HU ☐

1. Does the media or any publicity make you further aware of breast cancer?

- Yes ☐
- No ☐

1. How serious do you rate breast cancer as a disease?

- Not too serious ☐
- Serious ☐
- Extremely serious ☐
- Treatable ☐
- Life-threatening ☐

1. How likely is it that the following would prevent me from having a mammogram? *Cross (X) the most applicable box*

VL=Very Likely L= Likely M=Maybe U=Unlikely HU=Highly Unlikely

- Unnecessary radiation exposure

VL ☐ L ☐ M ☐ U ☐ HU ☐

- Time consuming

VL ☐ L ☐ M ☐ U ☐ HU ☐

- Painful

VL ☐ L ☐ M ☐ U ☐ HU ☐

- Too old

VL ☐ L ☐ M ☐ U ☐ HU ☐

- Too young

VL ☐ L ☐ M ☐ U ☐ HU ☐

- Something negative may be discovered

VL ☐ L ☐ M ☐ U ☐ HU ☐

- I do not know how to book a test

VL ☐ L ☐ M ☐ U ☐ HU ☐

- It is too difficult to get to the clinic

VL ☐ L ☐ M ☐ U ☐ HU ☐

- I don’t know what will be done during the test

VL ☐ L ☐ M ☐ U ☐ HU ☐

- The staff may not treat me well

VL ☐ L ☐ M ☐ U ☐ HU ☐

- It is too expensive

VL ☐ L ☐ M ☐ U ☐ HU ☐

- My doctor does not suggest it to me

VL ☐ L ☐ M ☐ U ☐ HU ☐

- It is embarrassing

VL ☐ L ☐ M ☐ U ☐ HU ☐

- It would interfere with my daily activities

VL ☐ L ☐ M ☐ U ☐ HU ☐

*Thank you very much for completing the questionnaire.*
